# Long-Term Immunological Alertness and Response to COVID-19 Vaccination—Conditions for Prevention in Early Palliative Oncological Care Patients

**DOI:** 10.3390/vaccines12030299

**Published:** 2024-03-13

**Authors:** Peter Priester, Miroslav Fajfr, Veronika Molnarova, Radek Sleha, Sylva Janovska, Pavel Bostik, Stanislav Filip

**Affiliations:** 1 Department of Oncology and Radiotherapy, University Hospital Hradec Kralove, 500 05 Hradec Kralove, Czech Republic; peter.priester@fnhk.cz (P.P.); veronika.molnarova@fnhk.cz (V.M.); 2 Institute of Clinical Microbiology, University Hospital Hradec Kralove, 500 05 Hradec Kralove, Czech Republic; miroslav.fajfr@fnhk.cz (M.F.); pavel.bostik@fnhk.cz (P.B.); 3 Institute of Clinical Microbiology, Faculty of Medicine in Hradec Kralove, Charles University, 500 03 Hradec Kralove, Czech Republic; radek.sleha@unob.cz; 4 Department of Epidemiology, Military Medical Faculty, University of Defense, 500 01 Hradec Kralove, Czech Republic; sylva.janovska@unob.cz

**Keywords:** palliative oncological care, COVID-19 vaccine, anti-S IgG, VNT, inpatient care

## Abstract

Aside from the general population, the COVID-19 pandemic has also affected a group of patients in palliative oncology care. In this study, long-term immune responses against SARS-CoV-2 after vaccination were monitored in a cohort of patients in palliative oncology care. This non-randomized, prospective, and open-label pilot study recruited patients from the Palliative Oncology Program and included 147 patients, of which 80 were females (54.4%) and 67 males (45.6%). The overall evaluation included current health status, SARS-CoV-2 anti-S IgG titer, and neutralizing antibodies using the SARS-CoV-2 virus neutralization test (VNT). Anti-S IgG antibody analysis revealed high (H) antibody levels in 35.7% (*n* = 10) and very high (VH) levels in 39.3% (*n* = 11) of patients after the second vaccination dose. Similarly, after the third dose, H was found in 29.6% (*n* = 32) and VH in 55.5% (*n* = 60) of patients. High and very high anti-S IgG antibody levels were consistent with high VNT titers (>2560) and H antibody levels in 17.1% (*n* = 12) or VH in 82.9% (*n* = 58) of patients. Patients with two or more doses showed H and VH antibody levels at a median of 451 and 342 days after vaccination, respectively. In this clinical trial, patients showed high and very high levels of anti-S IgG antibodies over a longer period of time. These patients did not show reduced immunological responses to the COVID-19 vaccine challenge. We can assume that prevention through vaccination can reduce the risk of complications or death from COVID-19 in patients in early palliative oncology care.

## 1. Introduction

The COVID-19 pandemic continues to vary in intensity and periods of peak activity, raising valid concerns regarding the quality and accessibility of healthcare. As an added complication, the pandemic affected several patient groups afflicted with serious illnesses, including cancer. Cancer patients are at higher risk of developing severe COVID-19 because of aggressive therapy, e.g., chemotherapy, radiotherapy, targeted therapy, immunotherapy, and their combinations. These treatments have a number of side effects, which can negatively affect the immune system, and, thus, this induced immunocompromised state poses an increased risk of infection [1,2].

Advanced cancer patients are kept under palliative care, which has specific parameters dictated by the nature of the disease, making a clear distinction between early, pre-terminal, and terminal palliative oncological care [3,4]. During the COVID-19 pandemic, a number of health, social, and psychological issues with a clear economic impact were made apparent [5], creating the need for proper palliative cancer care organization and planning. As a result, experimental and clinical research had to respond to the current requirements for COVID-19 diagnosis and treatment [2,5]. The COVID-19 pandemic is caused by one of the several variants of the coronavirus SARS-CoV-2 [2]. Infection with this virus activates both innate and adaptive immune systems, leading to a massive inflammatory response during the course of the disease and tissue damage, with serious long-term sequelae as well [6]. The unregulated inflammatory response can lead to local and systemic tissue damage unleashed by a cytokine storm. Strong humoral immune responses (B cells) are commonly caused by viral infections and represented by increases in serum levels of IgM, IgG, IgA, and neutralizing IgG antibodies (nAbs). In COVID-19, IgG, IgM, and IgA antibodies against SARS-CoV-2 can usually be detected in most patients along with viral RNA production [7,8].

SARS-CoV-2 stimulates rapid seroconversion in the IgG class of specific antibodies in symptomatic [9] and asymptomatic subjects [10], which lasts for a relatively long time and can be used as a long-term marker of previous SARS-CoV-2 infection [11]. Neutralizing antibodies are the best indicator of protection after vaccination and against reinfection [12,13]. In contrast to vaccination, several types of antibodies are produced against viral antigens during the infection, forming different IgA, IgM, and IgG isotypes that may enhance or alter the results of VNT levels [14]. Antibody level monitoring is thus a reliable tool for an assessment of both the COVID-19 infection history and vaccination response, as the antibodies can be present in the blood for a very long time (>10 months) [15]. The question, however, is whether cancer patients with a completed anticancer treatment can sustain these antibodies in the long term. It must be kept in mind that anticancer therapy has a wide range of adverse effects on the body [16]. Therefore, the influence of such therapy on the immune system (i.e., abscopal effect) cannot be ignored [17]. 

Termination of anticancer therapy in palliative oncological care patients can be caused by serious reasons, such as the series of adverse effects caused by the therapy. Therefore, it could be hypothesized that cancer patients are highly vulnerable to health complications and poor clinical outcomes after SARS-CoV-2 infection [16,17]. Overall, a three-dose vaccination series against COVID-19 was recommended for cancer patients, assuming that their immunological response could be reduced or impaired [18]. However, no adequate clinical study has been conducted in palliative cancer care patients to justify this measure. It must be noted that it might be difficult to describe the effects of vaccination in these patients, as it has been suggested that impaired immunity caused by an underlying malignancy or anticancer systemic therapies may result in lower antibody titers after vaccination and a possible risk of breakthrough infection [18,19]. Due to the high morbidity and mortality rates from COVID-19 among cancer patients, the benefits of vaccination are likely to outweigh the risks related to adverse events [19], more so because cancer patients often have worse outcomes in COVID-19 infection [20].

The research presented herein evaluated the long-term effects of preventive COVID-19 vaccination in palliative cancer care patients. The observations support the need for a COVID-19 vaccination plan for disease prevention in such patients and reveal key aspects of their immune response.

## 2. Methods

### 2.1. Design

This non-randomized, prospective, and open-label pilot study recruited patients from the Palliative Oncology Program in the Complex Oncology Center of the University Hospital Hradec Kralove. 

### 2.2. Setting

The clinical study and patient enrollment took place at the abovementioned clinic from September 2021 to June 2023. The patients were duly informed during their first visit of informed consent. 

Inclusion criteria: Patients (≥18 years) with a completed antitumor treatment in palliative oncology care (for >1 month), with a performance status (PS) better than 3 (PS 0–3) were recruited into the study. These patients received at least one COVID-19 vaccine dose less than 3 weeks prior to the start of the study and had a negative PCR test not older than 72 h. Patients who received 1–4 doses of vaccine, and with the last vaccination dose administration more than 35 days ago. were included.

Exclusion criteria: Patients undergoing palliative chemotherapy, radiotherapy, immunotherapy, or biological therapy were excluded, as well as those with clinical manifestations of other infectious diseases. The patients under a regimen of antibiotics, antivirals, or antifungals within the preceding week were not included. Neither were those patients included who received other vaccines (e.g., influenza, tetanus, hepatitis A or B, and others) within a period of 3 months prior to the start of the study. The patients with duplicate cancer or in another supportive program were not included either.

### 2.3. Participants

The study comprised 147 patients, 80 of which were females (54.4%, $\bar{x}$ = 68.2 years of age, range 39–98 years) and 67 males (45.6%, $\bar{x}$ = 9.4 years of age, range 22–94 years) (Table 1). In our study, during the period of inclusion of patients in the palliative oncology care program, there was metastatic involvement of the liver in 21% of patients, metastatic involvement of the lungs in 4%, combined metastases of the liver and lungs in 3.4%, and metastatic involvement of the bone in 2% of patients.

### 2.4. Serum Antibody Determination

Patient serum samples were aliquoted and used for the analysis of antibody titers in the laboratory of the Institute of Clinical Microbiology, University Hospital in Hradec Kralove. The quantitative CMIA kit SARS-CoV-2 IgG II QUANT for anti-S IgG (Abbott GmbH, Sligo, Ireland) was used. The analysis was performed according to current SOPs and manufacturer-specified procedures using the Abbott^®^, Architect i2000SR (Abbott Laboratories, Abbott Park, IL, USA) device. The results of the assays are expressed as AU/mL anti-S IgG antibodies. The antibody levels were categorized as negative (N) for values 0–50 AU/mL; low (L) for 51–1000 AU/mL; high (H) for 1001–10,000 AU/mL; and very high (VH) for 10,001–40,000 AU/mL for further analysis.

### 2.5. Virus Neutralization Assay

The virus neutralization assays were performed in the BSL3 laboratory facility of the Faculty of Military Health Sciences, University of Defense in Hradec Kralove. The SARS-CoV-2 variant B.1.258 was propagated using the VERO CCL81 cell line, and then used to determine the neutralization antibody titers. Briefly, the patient serum samples were heat-inactivated for 30 min at 56 °C. The samples were serially diluted (starting at 1:20) in DMEM and placed in 96-well plates. The samples were then mixed with an equal volume of the viral solution (2000 TCID50/mL) and incubated for 1 hour at 37 °C/5% CO_2_. After this period, 100 µL of the samples were placed in duplicate in 96-well plates containing a semi-confluent monolayer of Vero cells (one of them serving as a negative control) and incubated at 37 °C/5% CO_2_. After 3 days, the virus-neutralizing antibody titer (VNT) was determined as the highest serum dilution preventing infection in duplicate wells.

### 2.6. Data Collection

The patient cohort was divided as follows: COVID-19 standard vaccination (post-vaccination reaction); COVID-19 reinfection after vaccination, clinically confirmed, and cured; two COVID-19 vaccination doses, number of days since vaccine administration, antibody titration, and immune response; three COVID-19 vaccination doses, number of days since vaccine administration, antibody titration, and immune response; and four COVID-19 vaccination doses, number of days since vaccine administration, antibody titration, and immune response. The obtained results were shown and verbally interpreted to the patients during their next visit with a recommendation of further steps for prevention.

### 2.7. Data Analysis

The obtained data were evaluated using descriptive statistics depending on the nature of the data, i.e., counts and percentages for categorical characteristics and median and interquartile range (IQR). The data were analyzed using IBM SPSS Statistics for Windows v22.0 (Armonk, NY, USA: IBM Corp). The GraphPad Prism 9 software (version 9.40, GraphPad Software Inc., San Diego, CA, USA) was utilized for graphical outputs and basic statistical evaluation. Categorical variables of the number of vaccination doses and post-vaccination COVID-19 were compared using Fisher’s exact test. The normality evaluation was performed using the Anderson–Darling test and Shapiro–Wilk test. Dunn’s multiple comparison test was employed for the statistical evaluation of results between cohorts with or without infection of SARS-CoV2.

### 2.8. Ethics

The present study was performed under the oversight of the State Institute for Drug Control in the Czech Republic (SIDC) and in accordance with the institutional Ethics Committee of the Faculty Hospital in Hradec Kralove, approval number 202109P03.

## 3. Results

### 3.1. Patients, Antitumor Therapy, and COVID-19 Vaccination

The patients’ medical history related to the anticancer treatment was taken into consideration during the initial examination to estimate the overall condition of patients according to WHO (PS). The cohort consisted mainly of patients with colorectal and breast cancers (Table 1). 

In this study, patients were treated with the third and higher lines of antitumor treatment. Thus, 9.5% (*n* = 14) of patients underwent chemotherapy alone and 4.8% (*n* = 7) of patients underwent radiotherapy alone. A combination of chemotherapy and targeted anticancer therapy was received by 50.4% (*n* = 74) of patients. Combined chemotherapy plus radiotherapy and targeted anticancer therapy were applied in 22.4% (*n* = 33) of patients, and 12.9% (*n* = 19) of patients received combination therapy plus immunotherapy. The patients were vaccinated as follows: 36.7% (*n* = 54) with the Comirnaty vaccine (Pfizer, BioNTech, Mainz, Germany) and 63.3% (*n* = 93) with Spikevax (Moderna, Amsterdam, The Netherlands). A total of 428 vaccine doses were administered. Two patients (1.4%) received one dose, 15.6% (*n* = 23) received two doses, 73.5% (*n* = 108) received three doses, and 9.5% (*n* = 14) received four doses. A survey on post-vaccination response revealed mild reactions in 20 cases (4.7%) after the first dose, 20 (4.7%) after two doses, 19 (4.4%) after the third dose, and 4 instances (0.9%) after four doses. No severe reactions were recorded, and neither were there any cases of vaccination-related hospitalizations. The initial anamnestic investigation included a careful evaluation of COVID-19-related clinical events as well as the post-vaccination frequency of the SARS-CoV-2 infection, recording any serological or PCR-confirmed cases. The patients with only one vaccination dose showed only a mild infection course and did not need any supportive or antiviral treatments. A similar observation was made in the patients after two, three, and four doses, who showed infection incidences of 57.1%, 53.4%, and 50%, respectively. However, the statistical evaluation showed no statistically significant difference. Overall, COVID-19 was diagnosed in 54.4% of patients throughout the study period (Table 2). None of them was hospitalized and no COVID-19-related death was recorded.

### 3.2. Antibody Analysis

The anti-S IgG antibody level analysis was performed as part of the outpatient check-up, revealing that each of the two patients in the single vaccine dose group showed either low (L) or high (H) levels of circulating antibodies. Of the 23 patients with two doses of the vaccine, H levels were noted in 35.7% of patients (*n* = 10) and very high (VH) in 39.3% of patients (*n* = 11). The best results were observed in patients with three vaccination doses (*n* = 103), where H and VH levels were recorded in 31.1% (*n* = 32) and 53.4% (*n* = 55), respectively. In patients with four vaccination doses 4 (booster) (H) was recorded at 35.7% (*n* = 5) and (VH) at 64.3% (*n* = 9) (Table 3). 

**Figure 1:**
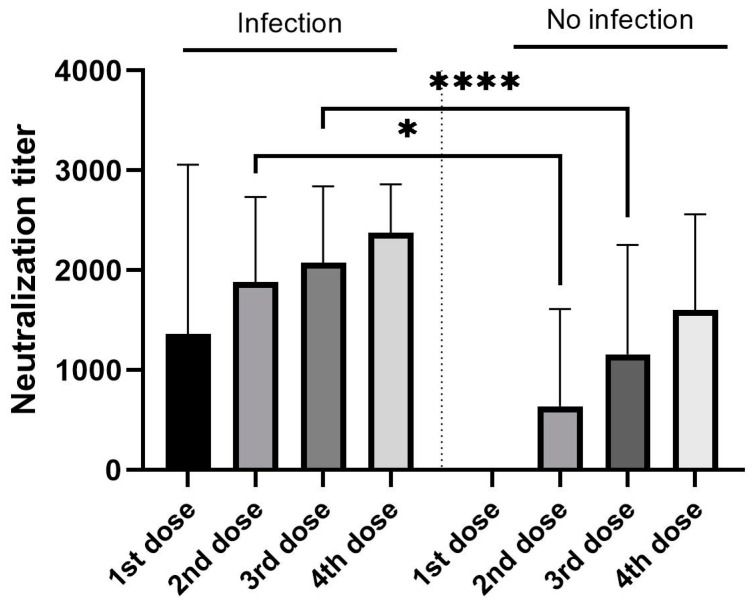
Statistical evaluation of VNT titer correlation to vaccine dose numbers and post-vaccination COVID-19 infection. Dunn’s multiple comparisons test was used (* *p* = 0.0472, **** *p* = 0.0001).

The VNT tests showed that H and VH levels of anti-S IgG antibodies were consistent with the high VNT titer results (>2560) in 17.1% (*n* = 12) of H patients and 82.9% (*n* = 58) of VH (Table 4). The association between vaccine dose numbers and VNT titers showed increasing VNT titer levels associated with an increasing number of vaccines, but when the statistical evaluation using Dunn’s multiple comparisons test was performed, no statistical significance was found (*p* value from 0.4755 to >0.9999). Then, the possible impact of post-vaccination COVID-19 infection on the VNT titer was evaluated. The significantly higher titers were found in those patients, who experienced COVID-19 after two (*p* = 0.0472) and three (*p* = 0.0001) vaccine doses (Figure 1).

The level and circulating time of anti-S IgG antibodies were also monitored in the patients after the application of the last vaccination dose (*n* = 147), showing that those who received at least two or more doses of the vaccine maintained H levels of antibodies for a median 451 days, and VH levels for a median 342 days (Table 5). 

## 4. Discussion

The COVID-19 pandemic raised awareness of both the indication and use of vaccines as a preventive measure. Despite the known outcomes of this pandemic, little attention was paid to high-risk patients. Cancer patients belong to this group, especially those in palliative care. Therefore, the acquisition of additional data on the effectiveness of COVID-19 vaccines is not only required but vital in the context of active cancer therapy. In this regard, some studies suggest that cancer patients possess increased complication and mortality risks related to COVID-19, e.g., a 30% mortality rate for hospitalized COVID-19 cancer patients compared to 21% for non-cancer patients [21,22,23,24]. The need for prevention remains a worrisome issue for palliative oncological care patients, which may be assuaged through newly developed therapeutic strategies for severely ill individuals. However, these may also present a prognostic challenge, especially for those patients previously ineligible for treatment due to age or present comorbidities [25]. Needless to say, palliative cancer care patients should be considered a high-priority COVID-19 vaccination subgroup [26,27], seeking in this manner to ameliorate the risk of death from COVID-19.

It is clear that the required, and very demanding, treatment places cancer patients at high risk, more so because the disease itself has a large impact on their immune system [28]. Therefore, it was widely assumed that the COVID-19 pandemic would have a heavily adverse effect on these patients. Further, it was also unclear how or if vaccination would protect them or what effect it could have on their overall survival [29,30]. Although some clinical studies have deemed COVID-19 vaccination as beneficial [22,23,24], the issue remains complicated for palliative oncological care patients. 

The adequate results of anticancer treatment often shift the timing for palliative care until the patient exhibits a good condition, i.e., performance status (PS) of 0–2, which qualifies them for early palliative oncological care [31,32]. Palliative cancer care outpatients have a clear reason for the requirement of COVID-19 vaccination. Therefore, the present study determined the relevance of COVID-19 testing in these patients of specific IgG antibodies, their circulating time, and VNT titers post-vaccination, thus correlating long-term immunological preparedness and response to COVID-19 vaccination and/or infection. However, a clear distinction had to be made between COVID-19-related and cancer progression symptoms [33]. The latter included the clinical manifestations of cancer therapy, in particular of the immuno- and targeted anticancer therapy [34]. The results have hereby shown partial support for other clinical studies. The previous statement notwithstanding, the booster effect of viral infection must be considered in those patients, where an immunosuppressive effect due to treatment is expected [35,36]. Regardless, the analyses made in this study suggest that early palliative cancer care patients should take advantage of both preventive and therapeutic measures to attenuate the risk of SARS-CoV-2 infection.

The need for COVID-19 vaccination as a preventive measure has been previously suggested for cancer patients [22,23,24], leading to the design of this clinical study based on the objective examination of the patients, including physical, clinical, and laboratory analyses. The clinical study followed careful inclusion criteria for the patients and considered the results of a nationwide prospective seroconversion study (PROSECO), which researched the dynamics of the anti-SARS-CoV-2 IgG antibodies in the Czech population. This study reported a large seropositivity increment over time, i.e., 28% in October/November 2020, 43% in December 2020/January 2021, and 51% in February/March 2021 [35], suggesting a high activity of the COVID-19 pandemic during these periods. During the latter of these periods, this clinical trial was started. A total of 147 (22.9%) patients were included in the study, all of whom had completed active anticancer therapy for at least 1 month, and thus their health status was stable overall. This was arranged purposefully so that the adverse effects of anticancer treatment and their associated symptoms (e.g., fatigue, nausea, weakness, subfebrile, and dyspepsia, among others) were less frequent or absent, thus reducing bias or altered data input in the study. The patients in our study did not have serious clinical complications in the event of a disease with COVID-19, and, due to this, the application of drugs such as Molnupiravir, Paxlovid, and additional ones containing the substances casirivimab and imdevimab and others were not indicated. In addition, the patients in the study were in outpatient treatment and often the information about the COVID-19 disease was recorded with a delay.

The correct timing for COVID-19 vaccination during the anticancer treatment remains to be determined [37] and was not monitored in this clinical pilot study. However, proper awareness and documentation were kept on the plausibility of immunosuppression caused by anticancer therapy. The follow-up period began after the last vaccine dose was administered, as verified by the Ministry of Health of the Czech Republic under patient consent. Previous clinical studies have described elevated IgA and IgG antibody levels that were detectable after a 12-month follow-up period [38,39], whereas other studies report reduced antibody levels (~50%) within 6 months, becoming stable up to 12 months [40]. Similar observation has been made in cancer patients, who have shown decreased SARS-CoV-2 antibody titers during a similar period, but with the antibodies remaining present for up to 9 months after vaccination [41,42]. Similar results were found in this clinical study, in which the patients with at least two or more vaccination doses displayed high ($\tilde{x}$ = 451 days) and very high antibody titers ($\tilde{x}$ = 342 days), thus confirming that the immune response in palliative care patients was not compromised by the anticancer treatment. However, it is not possible to clearly separate the effects of COVID-19 infection after vaccination [43,44]. Even the patients with one to four vaccination doses independent of vaccine type experienced COVID-19 infection in at least 54.4% of cases during the course of this study (800 days). Importantly, however, no COVID-19-related deaths were recorded. The evaluation of post-vaccination COVID-19 infection showed higher VNT titers in this group of patients compared to the patients without the infection. The reason for these findings is in the immune booster after the COVID-19 infection, which led to increasing VNT titers. Interestingly, out of 67 patients with a serologically proven infection, 27 (40.1%) lacked anamnestic data on COVID-19 infection, meaning that they were completely asymptomatic. High levels of anti-S IgG antibodies were detected during the present study, which could be directly correlated with the high antibody titers revealed by the VNT tests (>2560). Our previous study of the Abbott^®^ (Sligo, Ireland) anti-S SARS-CoV-2 IgG CMIA antibody test and VNT titers showed their correlation as Spearman r = 0.7298 [45]. These results place special attention on the timing and inclusion of palliative cancer care patients in the COVID-19 vaccination schedule and prevention programs, which can attenuate the risk of COVID-19 complications and/or death. It is currently recommended that cancer patients be vaccinated in a regular manner [18,46,47]. The common acceptance of the COVID-19 vaccine is not universal, and several reports have described situations where people strongly refuse to be vaccinated [48,49]. A similar situation may also arise in cancer patients under or after treatment. Moreover, cancer patients often choose an outpatient status for early palliative oncological care, and, thus, their willingness to accept the vaccine must be objectively and responsibly considered according to the recommended healthcare standards.

## 5. Conclusions

Palliative oncological care outpatients, as a general rule, have been subject to an aggressive anticancer treatment, which also results in a wide range of symptoms related to both therapy and disease progression. Regardless, these patients have an excellent and long-term immunological response to vaccination, and few present significant repercussions post-vaccination. Furthermore, these patients displayed an adequate immunological response to COVID-19 vaccination, and no related deaths were recorded in the cohort. Prevention through vaccination significantly reduces the risk of complications or death from COVID-19 in early palliative oncological care patients. 

## Figures and Tables

**Table 1:** Participants in the clinical study.

Patients (*n* = 147)	
Female 54.4% (*n* = 80)	Average age 68.2 years (ranged 39–98 years)
Male 45.6% (*n* = 67)	Average age 69.4 years (ranged 22–94 years)
ECOG Performance Status Scale (PS)	PS 0–3; median 2
Diseases	
Colorectal cancer	24 (16.3%)
Breast cancer	23 (15.6%)
Urinary cancer	17 (11.6%)
Pancreatic cancer	14 (9.5%)
Head–neck cancer	10 (6.8%)
Ovarian cancer	10 (6.8%)
Liver cancer	9 (6.2%)
Stomach cancer	8 (5.4%)
Tumors of unknown origin	7 (4.8%)
Lung cancer	6 (4.1%)
Other tumors	19 (12.9%)

**Table 2:** Correlation of the number of vaccination doses and rates of COVID-19.

	COVID-19 Infection *			Fisher’s Test	Statistical Significance
Number of Vaccinations	Yes	Yes (%)	No		
One dose	2	100	0	0.5005	ns
Two doses	16	57.1	12	0.8343	ns
Three doses	55	53.4	48	0.7216	ns
Four doses	7	50.0	7	0.7828	ns
TOTAL	80	54.4	67		

* Association between the number of administered vaccine doses and COVID-19. The results show a higher percentage of infection in patients with only one dose (*n* = 147; follow-up 800 days). Statistical analysis (Fisher’s exact test) showed no significant differences between the number of vaccine doses and post-vaccination COVID-19 infection (ns—not significant).

**Table 3:** Number of vaccination doses and their correlation to anti-S IgG levels.

Number of Vaccination Doses *	One	Two	Three	Four
N (0–50 AU/mL)	0	3 (10.7%)	2 (1.9%)	0
L (51–1000 AU/mL)	1 (50%)	4 (14.3%)	14 (13.6%)	0
H (1000–10,000 AU/mL)	1 (50%)	10 (35.7%)	32 (31.1%)	5 (35.7%)
VH (10,001–40,000 AU/mL)	0	11 (39.3%)	55 (53.4%)	9 (64.3%)
TOTAL	2	28	103	14

* The correlation between the number of administrated vaccine doses and anti-S IgG levels (*n* = 147). The data show a higher correlation in patients with more than one vaccination dose. Categories: N—negative; L—low; H—high; VH—very high.

**Table 4:** Comparative analysis of VNT antibody titers and anti-S IgG levels.

Anti-S IgG Level	VNT Titer *								
	0	40	80	160	320	640	1280	2560	>2560
N (0–50 AU/mL)	4	0	0	0	0	0	1	0	0
L (51–1000 AU/mL)	3	7	4	5	0	0	0	0	0
H (1000–10,000 AU/mL)	0	2	4	1	3	7	16	3	12
VH (10,001–40,000 AU/mL)	0	0	0	0	1	2	12	2	58
TOTAL	7	9	8	6	4	9	29	5	70

* Correlation between SARS-CoV-2 anti-S IgG antibodies and VNT antibody titers (*n* = 147). Categories: N—negative; L—low; H—high; VH—very high.

**Table 5:** Correlation of the time elapsed since the last vaccination dose and anti-S IgG antibody levels.

Days after Vaccination *	N (0–50 AU/mL)	L (51–1000 AU/mL)	H (1000–10,000 AU/mL)	VH (10,001–40,000 AU/mL)
1–100	0	0	0	4
101–200	0	2	3	17
201–300	1	5	12	12
301–400	1	7	11	12
401–500	1	2	14	11
501–600	1	3	5	15
601–700	1	0	3	2
701–800	0	0	0	2
TOTAL	5 (3.4%)	19 (12.9%)	48 (32.7%)	75 (51.0%)

* Data show a dependence of SARS-CoV-2 anti-S IgG antibody levels on the time after the last vaccine dose (*n* = 147). Categories: N—negative; L—low; H—high; VH—very high.

## Data Availability

The data presented in this study are available on request from the corresponding author.
